# Tongue diagnosis indices for gastroesophageal reflux disease: A cross-sectional, case-controlled observational study

**DOI:** 10.1097/MD.0000000000020471

**Published:** 2020-07-17

**Authors:** Tzu-Chan Wu, Cheng-Nan Lu, Wen-Long Hu, Keng-Liang Wu, John Y. Chiang, Jer-Ming Sheen, Yu-Chiang Hung

**Affiliations:** a Department of Chinese Medicine, Kaohsiung Chang Gung Memorial Hospital and Chang Gung University College of Medicine; b Fooyin University College of Nursing, Kaohsiung; c Kaohsiung Medical University College of Medicine; d Division of Hepatogastroenterology, Department of Internal Medicine, Kaohsiung Chang Gung Memorial Hospital, Chang Gung University, College of Medicine; e Department of Computer Science and Engineering, National Sun Yat-sen University, Taiwan.

**Keywords:** Chinese traditional medicine, gastroesophageal reflux, tongue diagnosis

## Abstract

Traditional Chinese medicine tongue diagnosis can mirror the status of the internal organ, but evidence is lacking regarding the accuracy of tongue diagnosis to gastroesophageal reflux disease (GERD). This study was to investigate the association between GERD and tongue manifestation, and whether tongue imaging could be initial diagnosis of GERD noninvasively.

We conducted a cross-sectional, case-controlled observational study at Kaohsiung Chang Gung Memorial Hospital in Taiwan from January 2016 to September 2017. Participants aged over 20 years old with GERD were enrolled and control group without GERD were matched by sex. Tongue imaging were acquired with automatic tongue diagnosis system, then followed by endoscope examination. Nine tongue features were extracted, and a receiver operating characteristic (ROC) curve, analysis of variance, and logistic regression were used.

Each group enrolled 67 participants. We found that the saliva amount (*P* = .009) and thickness of the tongue's fur (*P* = .036), especially that in the spleen–stomach area (%) (*P* = .029), were significantly greater in patients with GERD than in those without. The areas under the ROC curve of the amount of saliva and tongue fur in the spleen–stomach area (%) were 0.606 ± 0.049 and 0.615 ± 0.050, respectively. Additionally, as the value of the amount of saliva and tongue fur in the spleen–stomach area (%) increased, the risk of GERD rose by 3.621 and 1.019 times, respectively. The tongue fur in the spleen–stomach area (%) related to severity of GERD from grade 0 to greater than grade B were 51.67 ± 18.72, 58.10 ± 24.60, and 67.29 ± 24.84, respectively.

The amount of saliva and tongue fur in the spleen–stomach area (%) might predict the risk and severity of GERD and might be noninvasive indicators of GERD. Further large-scale, multi-center, randomized investigations are needed to confirm the results.

Trial registration: NCT03258216, registered August 23, 2017.

## 1 Introduction

Gastroesophageal reflux disease (GERD) is an irritating and prevalent digestive disorder that affects patients of all ages. According to the Montreal consensus statement, GERD develops when the stomach's contents return into the esophagus, resulting in troublesome symptoms and/or complications.^[1]^ The typical symptoms of GERD include heartburn, regurgitation, and reflux chest pain syndrome. Extra-esophageal symptoms include coughing, hoarseness, asthma, chronic laryngitis, dyspepsia, nausea, or dental erosion.^[1,2]^ The prevalence of GERD was estimated to be 8.8% to 25.9% in Europe and 2.5% to 7.8% in East Asia.^[3]^ In Taiwan, the prevalence of GERD ranged from 3.9% to 25%.^[4]^ Furthermore, GERD affects 30% to 40% of the population of the United States, with annual health care payments of $12 billion and nearly $50 billion for those with suspected extra-esophageal reflux.^[5,6]^ Clinically, GERD can impact the patient's quality of life,^[7]^ quality of sleep, and personal working performance,^[8]^ and can even lead to mental disorders.^[9,10]^ Not only does GERD have a physiological and psychological influence, but it also places an economic burden on health care resources.^[11]^ Therefore, GERD is an important issue to be addressed.

According to the guidelines of the American College of Gastroenterology,^[12]^ there is no gold standard method to diagnose GERD, but the diagnosis is usually made based on the disease's clinical manifestation and objective testing with endoscopy, ambulatory reflux monitoring, or the patient's response to acid-suppressive therapy. A 6- to 8-week course of empiric proton-pump inhibitor (PPI) therapy, known as the PPI trial, may confirm the presence of GERD when patients have the typical symptoms.^[13]^ However, this approach has some limitations: negative findings cannot rule out GERD, and PPI trial has a sensitivity of 78% and specificity of 54%.^[14]^

Esophagogastroduodenoscopy has been the primary tool to evaluate the different stages of disease severity in the esophageal and gastrointestinal mucosa for a long time.^[15]^ Endoscopy is an invasive and nonsurgical procedure with insertion of a long, thin, and flexible tube directly into the mouth, esophagus, and stomach to examine a patient's digestive tract in detail. Failure to respond to antisecretory therapy should evaluation with endoscopy. The role of endoscopy in the management of GERD is to a make positive diagnosis and rule out erosive esophagitis, peptic stricture, Barrett's esophagus, and esophageal adenocarcinoma. However, the majority of patients with the typical symptoms do not have erosion, thus limiting endoscopy as an initial diagnostic testing method.^[12,16]^ Ambulatory pH-impedance monitoring is an essential method to detect abnormal exposure esophageal acid and is indicated in patients who do not respond to PPI therapy and those with extra-esophageal symptoms, particularly those with non-erosive reflux disease preoperatively.^[17]^ Although this approach has excellent sensitivity (77–100%) and specificity (85–100%), it takes a long time to perform examination and high costs also limits the availability worldwide.^[18]^ Endoscopy may have a few potential complications including gut perforation, sedation effect, infection, or bleeding. Therefore, early non-invasive diagnostic or screening methods should be needed.

Tongue-based diagnosis, which is one of the diagnosis procedures in traditional Chinese medicine (TCM), plays an important role in the differentiation of symptoms and helps the physician to correctly diagnosis and properly treatment. The internal organs connect to the tongue through meridians; therefore, the tongue can mirror the status of the body and flow of qi and blood, and even help determine the severity of disease.^[19]^ However, the diagnosis method using such an observation often depends on the physician's subjective judgment and personal experience, as well as environmental factors. To overcome these limitations, many computerized tongue analysis systems have been developed to diagnose the condition of the tongue objectively and quantitatively.^[20]^ The automatic tongue diagnosis system (ATDS) is a computerized analysis system composed of image capturing, color calibration, tongue segmentation, and feature analysis. The intra-agreement of ATDS was proven to be significantly higher than the TCM doctors’ opinions alone, and there was moderate inter-agreement between ATDS and TCM doctors’ opinions.^[21]^ ATDS not only provides objective information about the tongue, but it also acts as an education tool for medical students who are learning how to diagnose the condition of the tongue.^[22]^ The computer-aided diagnosis might help the development of artificial intelligence with machine learning and deep learning in the future.

Several studies have been conducted to evaluate the relationship between tongue imaging findings and diseases such as rheumatoid arthritis,^[23]^ breast cancer,^[24,25]^ metabolic syndrome,^[26]^ and dysmenorrhea.^[27]^ However, to the best of our knowledge, no study has investigated the relationship between TCM tongue manifestations and GERD using ATDS. Therefore, the aims of the present study were to

1. realize the tongue manifestations of patients diagnosed with GERD using this objective computerized tongue analysis system and
2. investigate the possible association between GERD and the condition of the tongue, and further
3. to provide valuable information helping clinical doctors to initial diagnosis of GERD noninvasively.

## 2 Methods

### 2.1 Trial registration and ethics approval

This research study was approved by the institutional review board of the Chang Gung Medical Foundation (IRB no. 104–4725B) and registered at ClinicalTrials.gov (identification number NCT03258216). This study was performed in accordance with the ethical principles of the Declaration of Helsinki. The participants provided informed consent to participate. All authors had access to this study data and reviewed and approved the final manuscript.

### 2.2 Participants

We recruited outpatients from the Department of Hepatogastroenterology and the healthcare center of Kaohsiung Chang Gung Memorial Hospital in Taiwan, from January 2016 to September 2017. We included both men and women over 20 years old who met the criteria to undergo endoscopy, agreed to participate, and provided written informed consent. The criteria to perform endoscopy include patients who have complicated symptoms, or fail to response of antisecretory medical therapy, or participants who plan to self-paid physical examination. We excluded patients with hypertension, diabetes mellitus, hepatitis, or other systemic diseases, those who were pregnant, those who had an acute infection or cognitive impairment, those who were unable to protrude their tongue stably, and those who had a risk of temporomandibular joint dislocation.

### 2.3 Study design

**Figure 1:**
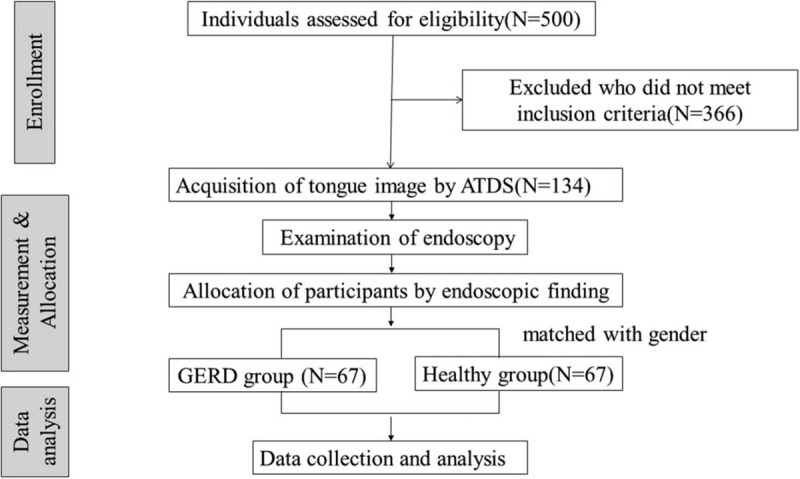
Flow chart of the study's design.

We conducted a cross-sectional, case-controlled observational study,^[28]^Figure 1 presents a flow chart of the study's design. After acquiring written informed consent from the patients, we collected their basic data, including sex, age, and medical history. All eligible patients will be then instructed to avoid food and liquid intake for 8 h before the examination of the ATDS and endoscopy at the next visit. At the second visit, the tongue's manifestations were obtained with ATDS under consistent environmental conditions and by the same highly educated operator. After the tongue images were captured, the participants then undergo endoscopy, which was performed by the professional gastroenterologist. Then, patients were allocated to one of two groups based on their clinical symptoms and endoscopic findings. Patients in the experimental group were symptomatic and diagnosed with erosive esophagitis using endoscopy; participants in the sex-matched control group were asymptomatic and had negative endoscopic findings. We analyzed the data of the subjects and the tongue features from the ATDS.

### 2.4 ATDS

**Figure 2:**
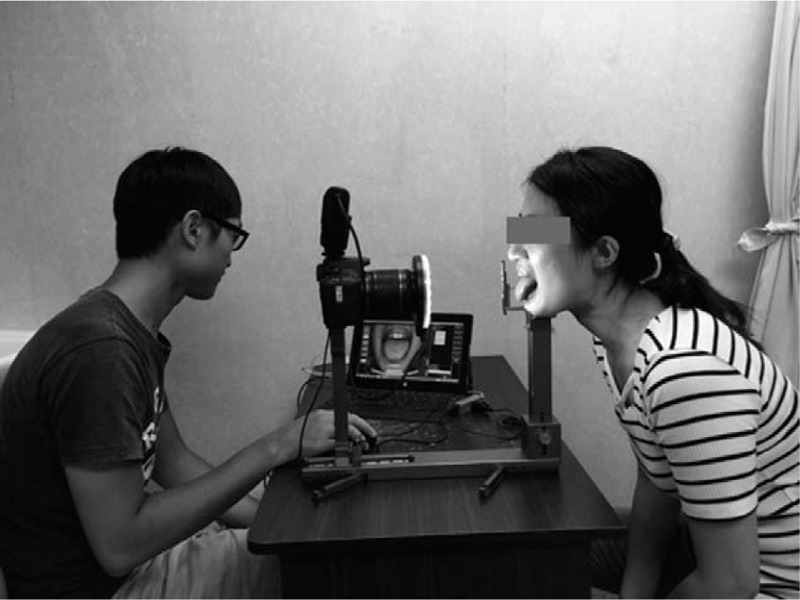
Operation of the ATDS. The ADTS was used to capture images of the tongue. The operator is shown on the left and the subject is shown on the right.

The ATDS was developed to capture tongue images and analyze the tongue's features. The components of the ATDS include a camera, light-emitting diode light, chin support, color bar, adjustment, and computer that can store and analyze images. The ATDS has three major functions: image capturing and color calibration, tongue area segmentation, and tongue feature extraction. A well-trained operator adjusted the chin support horizontally and vertically to capture an image of the whole tongue. Patient protruded his/her tongue stably for about 5 s without exerting force, allowing the operator to capture the image (Fig. 2). The ATDS can automatically correct any deviations in background light and color with the color bar. After the image was captured, the tongue region was identified and the irrelevant sections, including the teeth, lower facial portions, and background, were eliminated. Then, the features could be identified and extracted.

**Figure 3:**
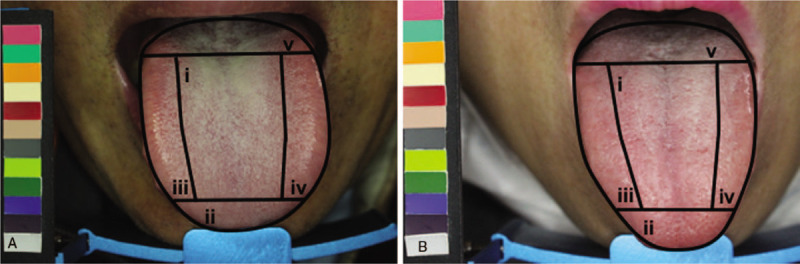
Comparison of the tongue images of patients with GERD and those of participants in the control group. (A) The patients in the GERD group had a higher amount of saliva, thicker tongue fur, and higher percentage of tongue fur in the spleen–stomach area than healthy people did. (B) Control group. The tongue was divided five segments corresponding to the internal organs according to the TCM theory: (i) the spleen–stomach area, (ii) the heart-lung area, (iii) the liver-gall-right area, (iv) the liver-gall-left area, (v) the kidney area.

The tongue image was further subdivided into five segments: the spleen–stomach, liver-gall-left, liver-gall-right, kidney, and heart–lung areas (Fig. 3), according to the TCM theory. Information on nine primary tongue features was extracted from the ATDS: tongue shape (small and thin, moderate, large, and fat), tongue color (slightly white, slightly red, red, dark red, and dark purple), tooth marks (number and covering area), tongue fissure (amount, covering area, and length), fur color (white, yellow, and dye), fur thickness (none, thin, and thick), saliva (total area and amount), ecchymosis (amount and covering area), and red dots (number and covering area).

### 2.5 Endoscopic findings

Endoscopic findings were recorded and interpreted by the gastroenterologist. After examining the endoscopic images, the doctor graded GERD lesions according to their severity using the Los Angeles classification,^[29,30]^ as follows: Grade A: one (or more) mucosal break no longer than 5 mm that does not extend between the tops of two mucosal folds; Grade B: one (or more) mucosal break longer than 5 mm that does not extend between the tops of two mucosal folds; Grade C: one (or more) mucosal break that is continuous between the tops of two mucosal folds but that involves <75% of the circumference of the esophagus, and Grade D: one (or more) mucosal break that involves at least 75% of the esophageal circumference.

### 2.6 Sample size

Power analysis can be used to calculate the minimum sample size required. With power = 0.8, alpha = 0.05, effect size convention *r* = 0.5, and an anticipated drop-out rate of 10%, the required sample size of each group was calculated to be 70, using G^∗^Power 3.0.1.0 software.

### 2.7 Statistical analysis

All statistical analyses were performed using the SPSS statistical package program (ver. 17.0, SPSS Inc, Chicago, IL). Chi-square tests were applied to test categorical data and analysis of variance (ANOVA) tests were used for continuous data. A logistic regression analysis was used to estimate the odds ratio and probability of a binary response. We applied ANOVA to realize the relationship between the severity of GERD and tongue features. To evaluate the sensitivity and specificity of the various tongue features that were used to diagnose GERD, we used a receiver operating characteristic (ROC) curve and area under the ROC curve (AUC). *P* values <.05 were considered statistically significant.

## 3 Results

### 3.1 Comparison of baseline characteristic

Between January 2016 and September 2017, we screened 444 participants for eligibility, of whom 67 diagnosed GERD. After adjusting for sex, the GERD and control groups included 67 participants with 30 men (45%) and 37 women (55%) each. The general characteristics of the participants in the GERD and control groups are presented in Table 1. The average age (mean ± SD) of the patients in the GERD group was 56.61 ± 13.40 years and that of participants in the control group was 53.84 ± 12.01 years. There were no significant differences between the two groups with respect to the baseline characteristics.

**Table 1:**
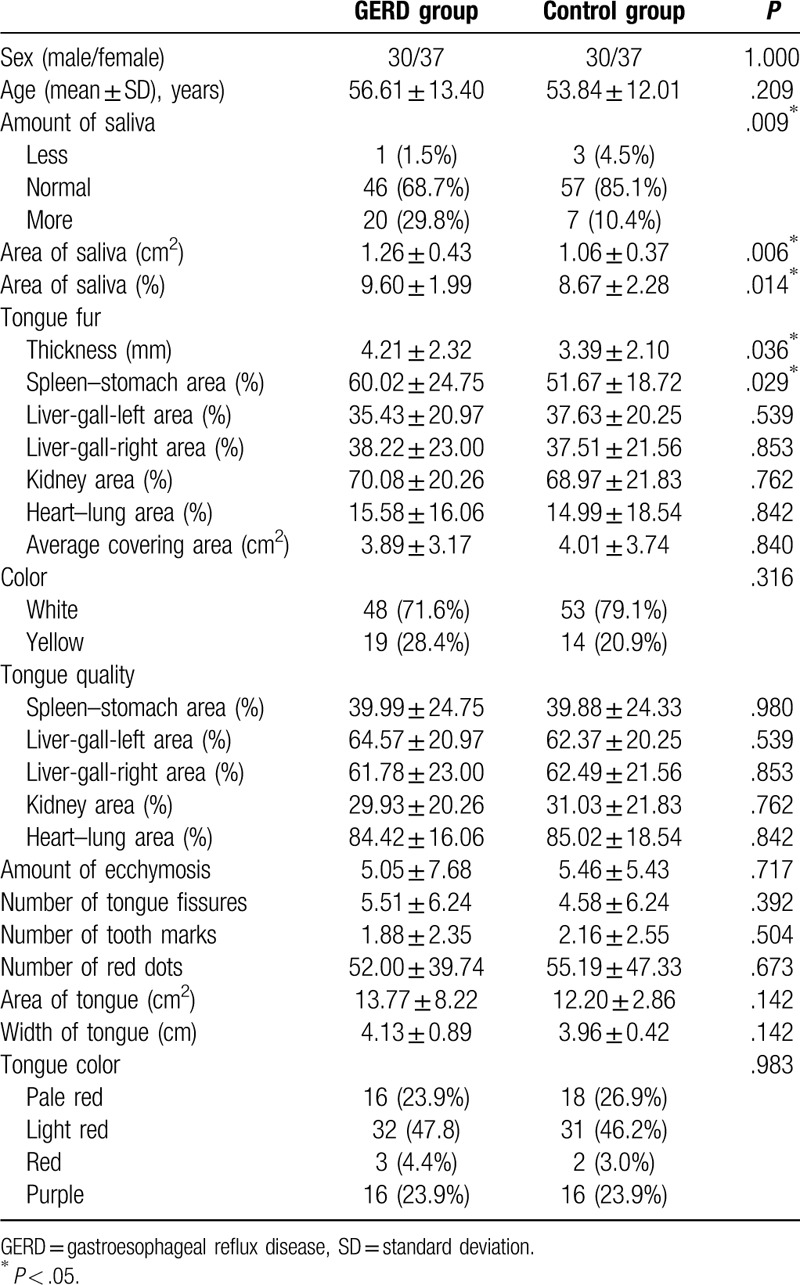
Characteristics of participants in the GERD and control groups.

### 3.2 Features seen on tongue imaging

ATDS could extract and analyze tongue features including the tongue's shape and color, tooth marks, tongue fissures, fur color, fur thickness, saliva, ecchymosis, and red dots. We found there was a greater amount of saliva, thicker tongue fur, especially the percentage of tongue fur in the spleen–stomach area in GERD patients than in the control participants (Table 1, Fig. 3). The amount and area of saliva were significantly larger in patients with GERD than in the control participants (amount of saliva, *P* = .009; area of saliva, *P* = .006). Furthermore, the tongue's fur was significantly thicker in patients with GERD than it was in the control participants (*P* = .036), especially the percentage of the tongue fur in the spleen–stomach area (*P* = .029). However, there were no significant differences between participants in the two groups regarding the tongue's color, fur color, tooth marks, ecchymosis, and red dots.

### 3.3 The diagnostic accuracy of tongue imaging

**Figure 4:**
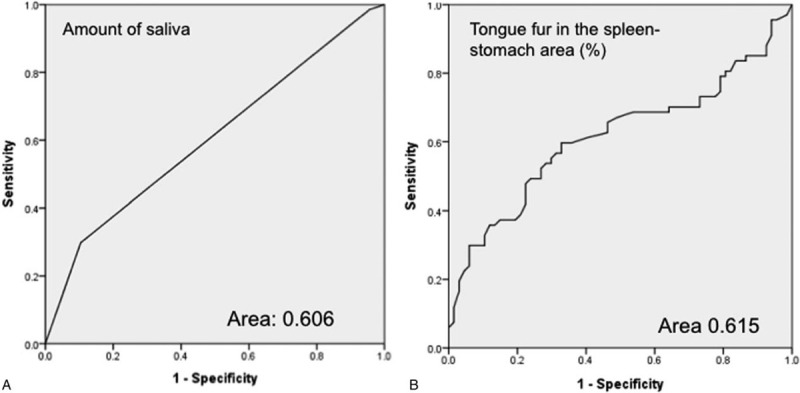
The ROC curve for the amount of saliva and tongue fur in the spleen–stomach area (%) for detecting GERD. (A) The amount of saliva. Area 0.66 ± 0.049; *P* = .034; 95% CI = 0.510–0.702. (B) Tongue fur in the spleen–stomach area (%). Area 0.615 ± 0.050; *P* = .021; 95% CI = 0.518–0.713.

Among the tongue features, there were significant differences in the amount of saliva, total area of saliva, thickness of the tongue's fur, and tongue fur in the spleen–stomach area (%). Therefore, the diagnostic accuracy was analyzed using a ROC curve and AUC (Fig. 4). The AUC for the amount of saliva was 0.606 ± 0.049 (95% CI, 0.510–0.702; *P* = .034), and that for the tongue fur in the spleen–stomach area (%) was 0.615 ± 0.050 (95% CI, 0.518–0.713, *P* = .021).

### 3.4 The risk of GERD

As shown in Table 2, a logistic regression analysis was performed. During the examination of the tongue, as the value of the amount of saliva and tongue fur in the spleen–stomach area (%) increased by one point, the risk of GERD rose by 3.621 and 1.019 times, respectively, with statistical significance (95%CI, 1.493–8.784; *P* = .004 and 95%CI, 1.003–1.036; *P* = .022, respectively). While the tongue image with more saliva and tongue fur in the spleen–stomach area was inspected, the risk of GERD enhanced.

**Table 2:**
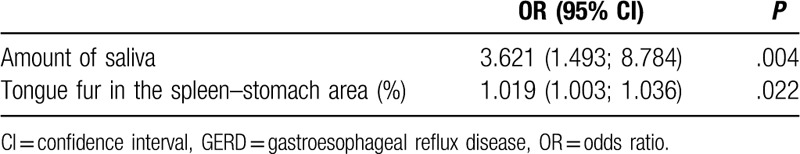
Comparison between the GERD and control groups using the logistic regression analysis.

### 3.5 The severity of GERD

**Figure 5:**
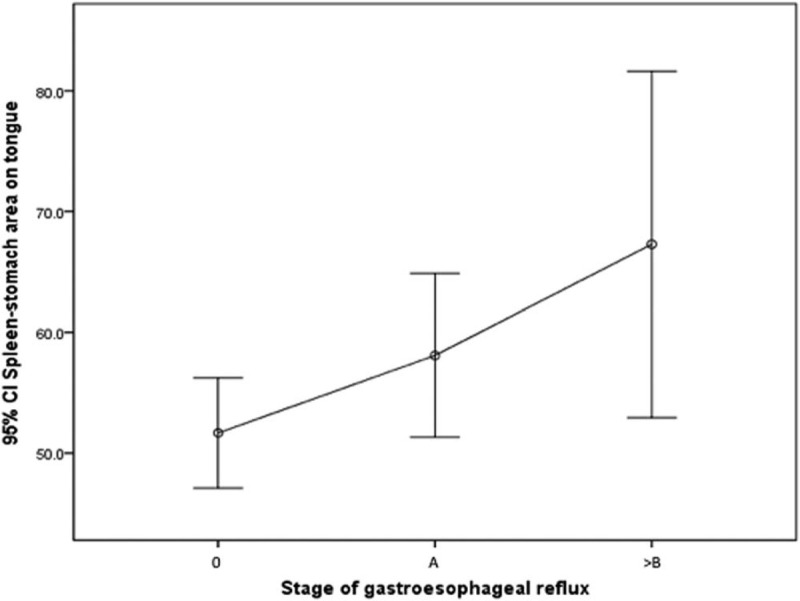
Relationship between spleen–stomach area on tongue and GERD stage. The data from stage 0 to Los Angeles grade A to greater than or equal to B were 51.67 ± 18.72 (95% CI = 47.11–56.24), 58.10 ± 24.60 (95% CI = 51.31–64.88), and 67.29 ± 24.84 (95% CI = 52.95–81.63), respectively (*P* value = .011).

We analyze the relationships between the tongue features and the severity of GERD by ANOVA, and the results revealed that the tongue fur in the spleen–stomach area (%) was statistical significance (*P* = .011) (Fig. 5). The data of the tongue fur in the spleen–stomach area (%) from stage 0 to greater than or equal to Los Angeles grade B were 51.67 ± 18.72 (95% CI = 47.11–56.24), 58.10 ± 24.60 (95% CI = 51.31–64.88), and 67.29 ± 24.84 (95% CI = 52.95–81.63), respectively (*P* = .011). That is, the higher the value, the greater the GERD stage was.

## 4 Discussion

We examined the disease manifestations on the tongues of patients diagnosed with GERD using an objective computerized tongue analysis system and investigated the possible association between GERD and the condition of the tongue as seen on imaging, using the TCM theory. The result revealed that a greater amount of saliva and thicker tongue fur especially the percentage of tongue fur in the spleen–stomach area indicated the presence of GERD. Acid regurgitation may increase the amount of saliva and thickness of the tongue's fur in the spleen–stomach area.

Inspecting images of the tongue is important in TCM. Tongue examinations are mainly performed to observe changes in the tongue's nature and coating. The normal tongue, which is mostly made of muscle, should be flexible. The normal finding of the tongue on imaging is a light red body with a thin white coating. Abnormal findings can help physicians to understand what is happening inside the patient's body. The tongue can be divided into five segments that correspond to the status of the body's system, including the heart–lung, spleen–stomach, kidney, and left/right liver-gall areas. Furthermore, according to the TCM theory, the tongue's fur, which refers to a fur-like substance covering the surface of the tongue, is created by “stomach-qi.” Therefore, the tongue, especially the spleen–stomach area, and tongue fur could reflect the status of the digestive system.

In this study, we found that the tongue images of patients with GERD tend to with more amounts of saliva and fur in the spleen–stomach area. According to the TCM theory, a thicker tongue coating with more saliva corresponds to phlegm and dampness which is related to obesity constitutional types. Interestedly, obesity is one of etiological factors of GERD.^[31]^ Several studies have proven that obesity and dietary habits are related to GERD.^[32,33]^ Weight loss and dietary modification also have been demonstrated to improve the symptoms of GERD.^[34,35]^ It has been recognized that GERD is one of the obesity-related comorbidities. Therefore, it might be the correlation between GERD and obesity through inspecting the tongue images.

ROC curve, or a ROC curve, is plotted by the true positive rate (sensitivity) against the false positive rate (1 − specificity) at various threshold settings. In addition to the endoscope, sensitivity and specificity combined ROC curve could increase the value of tongue diagnosis. The ROC curve is an effective method to assess the sensitivity and specificity of the tongue's features to diagnose GERD. The AUC is frequently used as a precision index, and a value larger than 0.5 indicates diagnostic accuracy. In our study, we examined the sensitivity and specificity of the amount of saliva and the percentage of tongue fur in the spleen–stomach area to diagnose GERD. The results demonstrated that these tongue features have the diagnostic ability and may provide early information about GERD. To the best of our knowledge, no studies have evaluated the diagnostic accuracy of tongue features to diagnose GERD.

Approximately 10% to 15% of patients of GERD might progress to major benign or precancerous complications, also called Barrett's esophagus, due to the severity of reflux into the esophagus and chronic injury can lead to malignant transformation.^[36]^ Affected patients have poorer health-related quality of life and higher economic burdens than healthy people do. Early diagnosis and treatment is needed. We found that the higher scores for the amount of saliva and the tongue fur in the spleen–stomach area (%) indicated a higher risk of disease and more severe GERD. Tongue-based diagnosis correlated positively with the results of endoscopy. Abnormal findings of non-invasive tongue imaging, such as the amount of saliva and tongue fur in the spleen–stomach area, may help clinicians before endoscopy is performed.

Few studies have discussed the connection between gastrointestinal disorders and tongue features such as the thickness of the tongue's fur,^[37]^ microbiota,^[38]^ or tongue temperature,^[39]^ but other features of the tongue were not mentioned. Sun et al found that changes in the metabolic components and micro-ecological indexes of the tongue's fur were associated with chronic gastritis,^[38]^ but the thickness or other features of the tongue was not mentioned. Kainuma et al concluded that the tongue body color of the middle area reflects acute change of gastric mucosa.^[40]^ Hu et al indicated that the tongue fur of patients with gastric cancer was significantly thicker than that of healthy control participants, and bacteria were associated with the appearance of tongue's fur.^[41]^ Similar findings were reported on colorectal cancer.^[42]^ However, the authors noted that the diagnostic sensitivity must be confirmed and more tongue features should be analyzed. Wang et al compared the tongue manifestations in patients with peptic ulcer disease before and after treatment. It concluded that the tongue's fur was markedly thinner, its color had changed to white, and engorged sublingual veins had improved after the ulcer healed.^[43]^

Based on the above studies, we can generally summarize that gastrointestinal problems are more or less associated with changes in the tongue's fur or spleen–stomach area. Further studies are needed to explore the differences of gastrointestinal disorders in tongue manifestations. Besides, the strength of our study is that computerized tongue analysis system was used to analyze the tongue objectively and quantitatively. It can assist physicians in interpreting medical images and capture quantitative information about facial features to improve the reliability and consistency of the diagnosis.^[44]^ Through the patient's symptoms and the change on the color and shape of the tongue, TCM physicians might realize the probability and severity of GERD before endoscope, especially for patient who is contraindicated of endoscope. Furthermore, even if patients have no symptoms, abnormal tongue findings might remind physicians of further evaluation.

Our study has several limitations. First, we did not evaluate the sublingual region. An inspection of the sublingual region would provide important information about the blood's circulation. However, we were unable to obtain information about the sublingual region because of instrument limitations. Second, the limited sample size of this study may have caused a bias due to variations in the population. Third, this study was designed as a single-center, cross-sectional study without randomization, blinding, or allocation concealment. Therefore, further large-scale, multi-center, randomized investigations are needed to confirm our results. In addition, the past history, underlying systemic diseases and other gastrointestinal diseases might be possible confounding factors. The participants with hypertension, diabetes mellitus, hepatitis, or other systemic diseases were excluded in the beginning. However, other gastrointestinal diseases cannot be ruled out so that further studies are needed.

In conclusion, this study demonstrated that the amount of saliva the tongue fur in the spleen–stomach area reflects the GERD. The TCM tongue diagnosis has clinical potential to predict the risk and the severity of GERD, even might be used as an indicator for diagnosing GERD. Therefore, our results might help physicians early diagnose GERD noninvasively.

## Acknowledgments

We appreciated the Biostatistics Center, Kaohsiung Chang Gung Memorial Hospital for statistics work. Besides, we would like to express our gratitude to the other members of the research team who participated in this study: Jun-Cheng Su and Ming-Zhi Lin.

## Author contributions

**Data curation:** Tzu-Chan Wu, Keng-Liang Wu.

**Formal analysis:** John Y. Chiang.

**Methodology:** Yu-Chiang Hung.

**Supervision:** Yu-Chiang Hung.

**Writing – original draft:** Yu-Chiang Hung, Tzu-Chan Wu.

**Writing – review & editing:** Yu-Chiang Hung, Cheng-Nan Lu, Wen-Long Hu, Jer-Ming Sheen.
